# When dystrophic plasma cells do not recognize themselves in the mirror

**DOI:** 10.1002/jha2.100

**Published:** 2020-10-09

**Authors:** Jean‐Baptiste Rieu, Aurore Perrot, Francois Vergez, Laetitia Largeaud, Jill Corre

**Affiliations:** ^1^ Haematology Laboratory Cancer University Institute of Toulouse–Oncopole Toulouse France; ^2^ Clinical Haematology Unit Cancer University Institute of Toulouse–Oncopole Toulouse France

**FIGURE 1:**
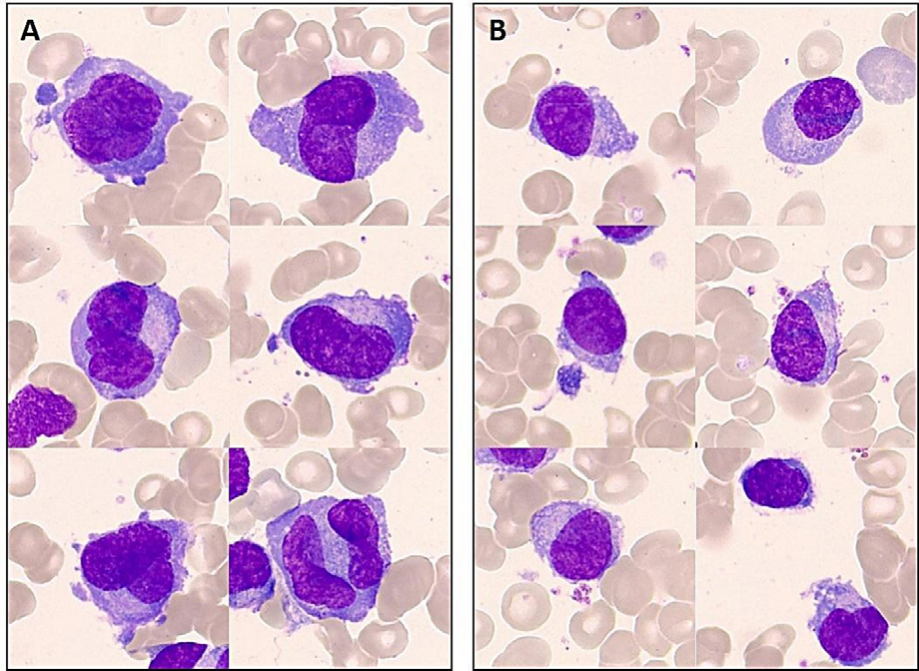
A, Dystrophic plasma cells of large size with irregular nucleus. B, Dystrophic plasma cells of small size with round nucleus. Bone marrow smear, May‐Grünwald‐Giemsa stain, 100× objective

A 71‐year‐old man consulted for a comprehensive health check. The patient was in perfect health (WHO performance status 0). Clinical examination was normal. The full blood count and differential count showed slight isolated anemia (Hb 116 g/L). Biochemical laboratory test results showed elevated creatinine (114 μmol/L) and urea (486 μmol/L), hyperproteinemia (99 g/L), and normal calcemia (2.34 mmol/L). Serum protein electrophoresis showed the presence of a monoclonal IgG kappa (29 g/L). Serum‐free light chain ratio (sFLCr) was 21. Bone marrow examination revealed numerous atypical cells of medium/large size, intermediate nuclear‐cytoplasmic ratio, irregular oval and occasionally bilobed nucleus, slightly decondensed chromatine with one or several inconspicuous nucleoli, and a basophilic cytoplasm that could be difficult to identify (Figure 1. A: May‐Grünwald‐Giemsa, 100× objective). However, some smaller cells with round eccentric nucleus simultaneously exhibit some of the above abnormalities and typical morphological features of plasma cells (Figure 1. B: May‐Grünwald‐Giemsa, 100× objective). This morphological continuum was clearly in favor of dystrophic plasma cells. With 69% of plasma cells in bone marrow, these results converged to the diagnosis of multiple myeloma despite the absence of CRAB criteria. Next‐generation sequencing (NGS) of plasma cells showed an hypodiploid molecular karyotype with t(11;14), monosomy 13, gain of chromosome 1q, loss of chromosome Y, and a clonal *IRF4* mutation. Del(17p) and t(4;14) were absent. Treatment with bortezomib, lenalidomide, and dexamethasone was initiated 1 month later.

Unlike normal and reactive plasma cells, which are usually easy to recognize, malignant plasma cells can present various and marked morphological abnormalities. Among them, irregular shape of the nucleus and/or multinuclearity (except binuclearity) may be confusing, whereas they must be considered as pathological in plasma cells. Fortunately, a continuum between atypical and classic plasma cells morphology is almost always observed in multiple myeloma and must be an argument to avoid misdiagnosis.

